# Microglia-Targeted Nanotherapeutics in Major Depressive Disorder: An Integrative Perspective on Neuroinflammation and Drug Delivery

**DOI:** 10.3390/pharmaceutics18010027

**Published:** 2025-12-25

**Authors:** Pablo R. da Silva, Nayana M. M. V. Barbosa, Joandra M. da Silva Leite, Larissa P. Alves, Jéssica C. de Andrade, Allessya L. D. Formiga, Ana Flávia C. Uchôa, Luiza C. D. Neri, Arthur Lins Dias, Adriana M. F. de Oliveira-Golzio, Francisco H. Xavier-Júnior, Ricardo D. de Castro, Cícero F. Bezerra Felipe, Marcus T. Scotti, Luciana Scotti

**Affiliations:** 1 Postgraduate Program of Dentistry (PPGO), Health Sciences Center, Federal University of Paraíba, João Pessoa 58051-900, PB, Brazil; pablorayff@ltf.ufpb.br (P.R.d.S.); rcastro@ccs.ufpb.br (R.D.d.C.); 2 Psychopharmacology Laboratory, Institute of Drugs and Medicines Research, Federal University of Paraíba, João Pessoa 58051-900, PB, Brazil; nayanamedeiros@ltf.ufpb.br (N.M.M.V.B.); jessicaca@ltf.ufpb.br (J.C.d.A.); luizaneri80@gmail.com (L.C.D.N.); arthurlinsd@gmail.com (A.L.D.); adrianamfoliveira@gmail.com (A.M.F.d.O.-G.); cicero@dbm.ufpb.br (C.F.B.F.); 3 Postgraduate Program in Natural Synthetic and Bioactive Products, Health Sciences Center, Federal University of Paraíba, João Pessoa 58051-900, PB, Brazil; mtscotti@gmail.com; 4 Department of Pharmaceutical Sciences, Federal University of Pernambuco, Recife 50670-901, PE, Brazil; joandramaisa@hotmail.com (J.M.d.S.L.); larissa.pereira@ufpe.br (L.P.A.); 5 Laboratory of Pharmaceutical Biotechnology (BioTecFarm), Department of Pharmaceutical Sciences, Campus Universitário I, Federal University of Paraiba, Castelo Branco III-Cidade Universitária, João Pessoa 58051-900, PB, Brazil; allessya.formiga@academico.ufpb.br (A.L.D.F.); anaflaviauchoauf@gmail.com (A.F.C.U.); fhxj@academico.ufpb.br (F.H.X.-J.); 6 Cheminformatics Laboratory, Institute of Drugs and Medicines Research, Federal University of Paraíba, João Pessoa 58051-900, PB, Brazil

**Keywords:** drugs delivery, neurological diseases, neuroinflammation, immunomodulation

## Abstract

Major depressive disorder (MDD) is a highly prevalent psychiatric condition characterized by complex neurobiological mechanisms, including oxidative stress and neuroinflammation, with microglial activation playing a key role in its pathophysiology. Conventional antidepressants, though widely used, often fail to achieve remission due to limited efficacy, adverse effects, and poor patient adherence. In this context, nanotechnology-based drug delivery systems have emerged as promising strategies to overcome pharmacological limitations, enhance blood–brain barrier (BBB) penetration, and target neuroinflammatory pathways. This narrative review explores the role of microglia as both mediators of neuroinflammation and potential therapeutic targets in MDD. We examine different nanocarriers and their ability to modulate microglial activation, promote a shift from a pro-inflammatory (M1) to an anti-inflammatory (M2) phenotype, and enhance antidepressant efficacy. Preclinical studies have demonstrated that nanoparticle-based systems not only improve drug bioavailability and brain targeting but also potentiate neuroprotective effects by reducing oxidative stress, promoting neurogenesis, and restoring synaptic plasticity. These findings highlight the potential of nanotechnology as a novel approach to precision neuropsychopharmacology. This review aims to provide an integrative perspective on how nanocarrier-based strategies targeting microglia could redefine future therapeutic paradigms for MDD.

## 1. Introduction

Depression is one of the leading causes of morbidity worldwide, with a high prevalence and a profound impact on quality of life. Prolonged stress is a major precipitating factor for severe depression and neurocognitive deficits, while affected individuals frequently develop comorbidities such as cardiovascular disease, diabetes, cancer, and obesity. Recent studies indicate that increased metabolic stress and accelerated cellular aging further compromise physical health, with oxidative stress, a key component of metabolic stress, being strongly implicated in the pathophysiology of depression [1,2].

Building on this clinical burden, mood disorders are among the most prevalent and severe psychiatric illnesses, encompassing unipolar depression (characterized by fluctuations between euthymia and depression) and bipolar disorder (which involves episodes of hypomania, mania, euthymia, and depression). According to the *Diagnostic and Statistical Manual of Mental Disorders* (DSM-5, 2013), major depressive episodes are associated with complex symptoms, including alterations in sleep, appetite, psychomotor activity, cognition, and mood [3].

In addition to their clinical heterogeneity, depressive disorders arise from a multifactorial interplay between genetic, environmental, and medical factors. Risk factors include a family history of depression (with approximately 35% of the risk being hereditary), childhood abuse and neglect, female sex, and recent exposure to stressors. Moreover, metabolic and autoimmune conditions significantly increase the risk of developing depressive symptoms [4].

Given this complexity, advances in neurobiology have been crucial in uncovering the underlying mechanisms of major depressive disorder (MDD). Beyond classical monoaminergic hypotheses, robust evidence highlights the role of neuroinflammation, particularly microglial activation, as a key driver of persistent and treatment-resistant depression [5,6,7]. Microglia, the primary immune cells of the central nervous system (CNS), maintain brain homeostasis through immune surveillance, clearance of cellular debris, and modulation of synaptic activity.

However, under chronic stress or systemic inflammation, microglia adopt a prolonged pro-inflammatory (M1) phenotype, releasing cytokines such as interleukin-1β (IL-1β), tumor necrosis factor-alpha (TNF-α), and interleukin-6 (IL-6), along with reactive oxygen species (ROS) [8]. These inflammatory mediators lead to neurochemical and neuroendocrine alterations, including glutamatergic dysfunction, neurotoxicity, reduced hippocampal neurogenesis, and impaired synaptic plasticity, all of which contribute to the persistence of treatment-resistant symptoms [9].

A central mechanism involved in this process is the activation of the NLRP3 inflammasome, responsible for the maturation of IL-1β and IL-18, thereby intensifying inflammatory responses associated with depressive-like behaviors. Microglia also modulate the kynurenine pathway by driving tryptophan metabolism toward neurotoxic metabolites, including quinolinic acid, which overstimulates NMDA receptors and contributes to excitotoxic neuronal damage. Furthermore, microglial dysfunction in the regulation of synaptic pruning, along with disrupted CX3CL1/CX3CR1-mediated communication with neurons, is linked to the synaptic deficits observed in major depressive disorder. Together, these interconnected mechanisms outline a neuroinflammatory framework in which microglial dysregulation plays a pivotal role in the pathophysiology of depression [6,7,8].

Supporting this inflammatory hypothesis, clinical and preclinical studies have consistently reported elevated levels of inflammatory markers, including C-reactive protein (CRP), IL-6, and TNF-α, in patients with treatment-resistant depression. This reinforces the urgent need for therapeutic approaches capable of modulating microglia-mediated neuroinflammation [7,10,11].

In this context, novel controlled drug delivery systems have emerged as a promising strategy to address the limitations of conventional antidepressant therapies, which are often associated with suboptimal efficacy, significant side effects, and poor patient adherence. Controlled and targeted drug delivery platforms have the potential to enhance therapeutic precision, reduce systemic toxicity, and improve clinical outcomes [12,13].

Furthermore, advanced delivery systems can be engineered for sustained or pulsatile drug release, decreasing dosing frequency and promoting better adherence. They also enable the co-delivery of multiple agents (e.g., antidepressants combined with anti-inflammatory or antioxidant drugs), supporting multimodal approaches to address the complex pathophysiology of treatment-resistant depression [14].

Among these innovative approaches, nanocarriers, such as polymeric nanoparticles, lipid-based systems, dendrimers, and exosomes, have gained attention for their ability to enhance bioavailability, achieve targeted brain delivery, and modulate the pharmacokinetics of therapeutic agents. These systems can cross the blood–brain barrier (BBB) via passive diffusion, carrier-mediated transport, or receptor-mediated transcytosis and can be functionalized to respond to pathological cues such as pH changes, oxidative stress, or inflammation [15].

Importantly, in the context of MDD, nanotechnology-based strategies hold promise not only for optimizing drug delivery but also for directly modulating microglial activity, a critical element in the neuroinflammatory pathways of depression [16]. Therefore, this review aims to explore the therapeutic potential of targeting microglia via nanotechnology as an innovative strategy to overcome neuroinflammation and advance the treatment of major depressive disorder.

## 2. Materials and Methods

In present study, we conducted a comprehensive narrative review of scientific literature sourced from reputable databases, including Google Scholar, ScienceDirect, and Pub Med. The search strategy employed a combination of relevant keywords such as “Major Depressive Disorder,” “Neuroinflammation,” “Microglia,” and “Nanotechnology” to ensure a broad yet focused examination of existing research. This narrative review aimed to explore how nanotechnological approaches can modulate microglial activity as a means to alleviate neuroinflammation associated with major depressive disorder (MDD).

Selected studies were critically analyzed to identify current advances in nanocarrier systems, such as polymeric nanoparticles, solid lipid nanoparticles, magnetic nanoparticles, dendrimers, and liposomes that demonstrate potential in crossing the blood-brain barrier and selectively targeting activated microglia. The review further assessed how these nanotechnological strategies influence inflammatory signaling pathways. The delivery routes and transport mechanisms explored include the use of nanocarriers capable of crossing the blood–brain barrier (BBB) via receptor-mediated transcytosis (RMT), adsorptive-mediated transcytosis (AMT), and carrier-mediated transport (CMT).

Additionally, approaches involving the pharmacological or genetic modulation of efflux transporters, such as P-glycoprotein (P-gp) and breast cancer resistance protein (BCRP), were considered to enhance brain bioavailability. Several delivery platforms were analyzed, including polymeric nanoparticles, solid lipid nanoparticles (SLNs), nanostructured lipid carriers (NLCs), dendrimers, magnetic nanoparticles, liposomes, exosomes, nanoemulsions, self-emulsifying drug delivery systems, and thermosensitive hydrogels, some of which are suitable for intranasal administration.

These technologies were evaluated for their ability to overcome the BBB, modulate inflammatory pathways, such as the NLRP3 inflammasome and Toll-like receptors (TLRs), and promote microglial polarization from the pro-inflammatory (M1) to the anti-inflammatory (M2) phenotype, highlighting their therapeutic implications in restoring neuroimmune homeostasis in MDD. Therefore, this study conducted an in-depth analysis of the mechanisms by which nanoparticle-based delivery systems interact with neuroimmune targets to reduce microglial-mediated neuroinflammation.

## 3. The Role of Microglia in MDD Pathophysiology

In the CNS, the maintenance of cerebral homeostasis is dependent upon a complex balance, which is primarily regulated by glial cells, particularly microglia. Among the various functions attributed to these cells, a key role is their ability to detect alterations in the neural environment and respond to threats to tissue integrity through receptors collectively known as the microglial sensome [17]. When homeostasis is disrupted, as in cases of infections, injuries, or chronic stress, glial cells undergo a phenotypic transformation, shifting from a quiescent state to a reactive profile. This transformation initiates a local-ized inflammatory response, a phenomenon referred to as neuroinflammation [18].

Microglial activation is triggered by danger signals such as pathogen-associated molecular patterns (PAMPs) and damage-associated molecular patterns (DAMPs), which are recognized by specialized receptors, notably the Toll-like receptors (TLRs). Upon activation, microglia undertake a range of essential functions, including the clearance of cel-lular debris, phagocytosis of dysfunctional synapses, removal of neurotoxic proteins, and active participation in synaptic pruning, a critical process during neural development and in synaptic remodeling throughout life [19].

Morphological and functional alterations of microglia in key brain regions, such as the prefrontal cortex (PFC), hippocampus (HIP), anterior cingulate cortex (ACC), and amygdala, have been consistently associated with the onset and persistence of MDD. Exacerbated microglial activation contributes to sustained states of neuroinflammation [20]. Microglial activation alternates between two functional states: M1 and M2. The M1 state is characterized by a pro-inflammatory response, whereas the M2 state is associated with anti-inflammatory and tissue-repair functions [21].

**Figure 1:**
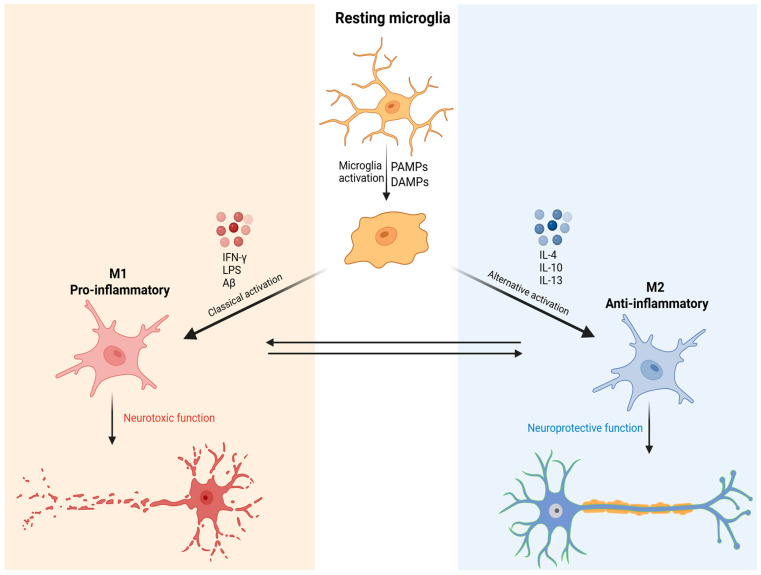
Schematic representation of microglial activation and functional polarization into M1 and M2 phenotypes. Resting microglia are activated by danger signals such as PAMPs and DAMPs. Classical activation induces the M1 phenotype, associated with pro-inflammatory cytokine release and neurotoxic functions. Alternative activation promotes the M2 phenotype, characterized by anti-inflammatory and neuroprotective functions. The plasticity between M1 and M2 states highlights the complexity of the immune response in the central nervous system. Created in BioRender. de Sá Braga Oliveira, A. (2025) https://BioRender.com/yrdy1ld (accessed on 11 December 2025).

During M1 activation, microglial cells increase in size, acquire an amoeboid mor-phology, and release pro-inflammatory cytokines to neutralize potential threats. This state is typically induced by stimuli such as interferon-gamma (IFN-γ), lipopolysaccharide (LPS), and beta-amyloid (Aβ). As a result, there is an elevated secretion of pro-inflammatory mediators, including TNF-α, IL-1β, ROS, and nitric oxide (NO). Conversely, the M2 phenotype is activated by anti-inflammatory cytokines such as interleu-kin-4 (IL-4), interleukin-10 (IL-10), and interleukin-13 (IL-13). The M2 profile is associated with the resolution of inflammation, tissue repair, and the promotion of neuroplasticity [11,22] (Figure 1).

**Figure 2:**
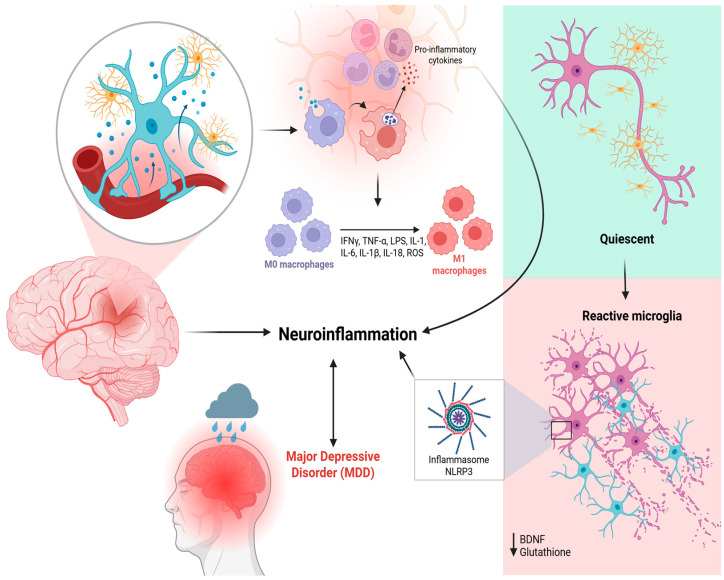
Schematic representation of microglial activation and its association with neuroinflammation and MDD. Under physiological conditions, microglia remain in a quiescent state, constantly surveying the neural environment. When cerebral homeostasis is disrupted, danger signals like PAMPs and DAMPs activate specialized receptors, particularly TLRs, leading to microglial phenotypic transformation. This activation contributes to the recruitment and polarization of M0 macrophages into pro-inflammatory M1 macrophages, which release inflammatory cytokines (TNF-α, IFN-γ, IL-1β, IL-6, IL-18) and ROS, perpetuating a neuroinflammatory state and depressive symptoms. This pro-inflammatory environment contributes to the activation of the NLRP3 inflammasome and a reduction in neuroprotective factors such as BDNF and glutathione. The resulting chronic neuroinflammation is implicated in the pathophysiology of MDD, as illustrated by the association between brain inflammation and depressive symptoms. Created in BioRender. de Sá Braga Oliveira, A. (2025) https://BioRender.com/yrdy1ld (accessed on 11 December 2025).

The current literature establishes a bidirectional relationship between mood and immune function, where depression may induce an inflammatory state, and conversely, systemic inflammatory processes can precipitate depressive symptoms [23]. This crucial link between neuroinflammatory processes and the pathophysiology of MDD is centrally mediated by aberrant microglial activation (Figure 2).

Under chronic stress, microglia shift from a homeostatic surveillance state (M0 phenotype Under chronic stress, microglia shift from a homeostatic surveillance state (M0 phenotype) to a predominantly pro-inflammatory profile (M1), characterized by Toll-like receptor (TLR3/TLR4) activation, upregulation of iNOS and CD86, and release of cytokines such as IL-1β, TNF-α, and IL-6.

These mediators trigger intracellular cascades, including NFκB and the NLRP3 inflammasome, fostering a neurotoxic environment that impairs synaptic plasticity and promotes neuronal apoptosis. In addition, hyperactivated microglia enhance the activity of indoleamine-2,3-dioxygenase (IDO), redirecting tryptophan metabolism toward the kynurenine pathway and increasing production of quinolinic acid, an NMDA receptor agonist with excitotoxic properties, at the expense of neuroprotective kynurenic acid (KYNA). This metabolic imbalance contributes to glutamatergic dysfunction and to the cognitive and affective deficits observed in MDD [24,25].

Another critical mechanism involves the interaction between microglia and the hy-pothalamic–pituitary–adrenal (HPA) axis. Elevated glucocorticoid levels in MDD patients, resulting from impaired negative feedback of glucocorticoid receptors (GRs), paradoxically act as a priming signal for microglia, rendering them more responsive to subsequent inflammatory stimuli. This priming induces persistent activation of the NFκB/NLRP3 pathway, perpetuating IL-1β and IL-6 release and establishing a vicious cycle of inflammation and neuroendocrine dysfunction [24,25,26].

Meanwhile, activation of the NLRP3 inflammasome is a central factor of neuroinflammation in MDD, and its sensitization is closely linked to reduced synthesis of BDNF, contributing to neurotrophic dysfunction and depressive symptoms. BDNF is essential for neuronal survival, synaptic plasticity, and hippocampal neurogenesis, all of which are commonly impaired in MDD [27].

Neurotrophic factors, particularly BDNF, have emerged as key elements in understanding the pathophysiology of MDD and mediating the response to antidepressant treatment. BDNF regulates neuronal survival, growth, and differentiation and is essential for maintaining synaptic plasticity and hippocampal neurogenesis—processes frequently impaired in individuals with depression [28].

At the same time, reactive astrocytes, which often show altered expression of markers such as GFAP, S100B, and AQP4, exacerbate chronic inflammation by releasing pro-inflammatory mediators and losing their metabolic and synaptic support roles. NLRP3 activation in astrocytes, especially under stress, further decreases BDNF levels and triggers caspase-1 and GSDMD-dependent pyroptosis, intensifying synaptic loss and neuronal damage. This dysfunctional interaction between microglia and astrocytes creates a self-sustaining cycle of inflammation, excitotoxicity, and neuroglial impairment that contributes to both affective and cognitive symptoms of MDD [24,25,26].

Additionally, disruption of neuron–microglia communication via reduced CX3CL1-CX3CR1 and CD200-CD200R signaling leads to excessive microglial activation, impaired synaptic pruning, and decreased synaptic density, ultimately disturbing dopaminergic and serotonergic circuits. The resulting shift toward a pro-inflammatory microglial state further aggravates corticolimbic dysfunction and perpetuates core depressive features such as anhedonia, fatigue, hopelessness, and cognitive decline [24,25,26].

## 4. Conventional Therapeutic Approaches and Their Limitations

Pharmacotherapy remains a key treatment for moderate to severe cases, and the past five decades have seen major advances in antidepressant development, particularly in selectivity and safety [29,30].

Pharmacological treatment of MDD is complex and multifactorial, requiring clinicians to have a detailed understanding of antidepressants’ mechanisms of action, pharmacokinetics, and safety profiles. Drug selection must be individualized, considering comorbidities, prior treatment response history, and risk of adverse effects. Studies such as those by Cipriani et al. [31] and Cui et al. [23], which comparatively analyze the efficacy and acceptability of various antidepressants, remain valuable tools for guiding evidence-based clinical practice.

Antidepressants exhibit a broad pharmacokinetic spectrum, influencing both clinical selection and dosing regimens. Hepatic metabolism via cytochrome P450 isoenzymes is a critical factor, as it affects drug interactions and individual therapeutic response. Pharmacogenetics, particularly involving CYP2D6, has also gained prominence in treatment personalization [28,32].

In this regard, selective serotonin reuptake inhibitors (SSRIs) are considered first-line drugs in the treatment of MDD due to their efficacy and a lower side effect profile compared to older antidepressants. The most frequently used drugs include fluoxetine, sertraline, paroxetine, citalopram, and escitalopram. SSRIs selectively block the serotonin transporter (SERT) in the synaptic cleft, thereby inhibiting reuptake of this neurotransmitter and increasing its postsynaptic availability [33].

However, sexual dysfunction, nausea, insomnia, weight gain, and, in some cases, serotonin syndrome when combined with other serotonergic drugs, are examples of adverse reactions that may hinder treatment adherence [27].

Another drug class used is serotonin-norepinephrine reuptake inhibitors (SNRIs), with venlafaxine, duloxetine, and desvenlafaxine being the main representatives. Their efficacy is related to the simultaneous action on SERT and norepinephrine transporters (NET), increasing synaptic levels of both neurotransmitters. Adverse reactions observed include dose-dependent hypertension (mainly with venlafaxine), nausea, dry mouth, sweating, and insomnia. Sexual dysfunction may also occur [34].

Tricyclic antidepressants (TCAs), represented by amitriptyline, nortriptyline, and imipramine, were the first antidepressants developed and, although effective, are currently less frequently used due to their less favorable safety profile. Their mechanism of action is based on the non-selective inhibition of serotonin (5-HT) and norepinephrine (NE) reuptake, as well as blocking cholinergic, histaminergic, and adrenergic receptors. Thus, the nonspecific reuptake blockade and interference with cholinergic, histaminergic, and adrenergic pathways result in more intense adverse reactions. Such reactions include sedation, orthostatic hypotension, constipation, urinary retention, blurred vision, and an increased risk of cardiac toxicity in cases of overdose [35].

Although less commonly used today, monoamine oxidase inhibitors (MAOIs) are indicated for treatment-resistant cases, with phenelzine, tranylcypromine, and moclobemide being the main representatives. These agents act by inhibiting the monoamine oxidase (MAO) enzyme, which is responsible for the degradation of monoaminergic neurotransmitters (5-HT, NE, and dopamine (DA)), thereby increasing their synaptic availability. Phenelzine and tranylcypromine have short half-lives but prolonged therapeutic effects due to irreversible MAO inhibition. Moclobemide, in contrast, is a reversible MAOI with a lower risk of dietary interactions. Excess neurotransmitter levels in the synaptic cleft are responsible for common adverse effects, including orthostatic hypotension, weight gain, insomnia, and the risk of hypertensive crisis when combined with tyramine-rich foods (the ‘tyramine effect’), as tyramine is also metabolized by MAO [36].

Despite the broad therapeutic arsenal currently employed for depression treatment, symptom persistence and failure to achieve remission remain common. Furthermore, antidepressants present several drawbacks. Modern agents such as SSRIs are associated with side effects such as nausea, headaches, insomnia, dizziness, sexual dysfunction, and weight gain. These symptoms frequently contribute to early treatment discontinuation by patients [37].

Recent studies have questioned the efficacy of antidepressants in mild to moderate depression, suggesting that the benefits may be limited and not superior to non-pharmacological interventions such as psychotherapy and lifestyle changes. Additionally, antidepressant discontinuation can be challenging due to withdrawal symptoms, which include dizziness, flu-like symptoms, sleep disturbances, and sensory alterations [38].

Given the presence of neuroinflammation, it has been questioned whether successful conventional treatment should consider reducing the inflammatory process. Although no currently approved antidepressant treatment was originally designed to modulate the immune response, there is evidence that conventional antidepressants exert significant anti-inflammatory effects. For example, SSRIs reduce levels of IL-1β, IL-6, and TNF. Conversely, other studies have shown that antidepressants such as SNRIs may induce the production of IL-6 and TNF [39,40,41].

These findings suggest that the effects of conventional antidepressants on cytokine regulation remain underexplored, although they are generally believed to shift the immune balance toward an anti-inflammatory profile. On the other hand, elevated levels of pro-inflammatory cytokines, in particular TNF, have been observed in treatment-resistant depressed patients, suggesting a negative correlation between therapeutic response and pro-inflammatory cytokine levels [5].

The interaction between neuroinflammation and psychiatric symptoms highlights the therapeutic potential of targeting inflammation in the treatment of depression. Anti-inflammatory agents, whether pharmacological interventions or lifestyle-based approaches (e.g., diet and exercise), are being explored as adjuncts to conventional antidepressant treatments, aiming to improve patient outcomes by addressing the underlying inflammatory processes. In this sense, neuroinflammation represents a promising avenue for understanding the biological basis of depression. As research continues to elucidate the mechanisms linking inflammation to mood regulation, there is growing hope for the development of more effective treatments targeting these pathways, offering relief to individuals affected by these widespread mental health conditions [42].

Considering this evidence, it becomes essential that future treatment strategies for persistent depression include agents with specific actions on CNS inflammatory mechanisms, particularly those that modulate microglial activity, such as pro-inflammatory cytokine inhibitors (e.g., anti-TNF-α), antioxidants, and glutamate receptor (NMDA) modulators. This represents a paradigm shift, integrating neuroimmunology into clinical psychiatry and opening new perspectives for managing treatment-resistant depressive disorders. Overall, as previously discussed, the molecular mechanisms underlying the pathophysiology of MDD identify microglial activation and the persistence of a neuroinflammatory state as critical therapeutic targets.

Nevertheless, although conventional antidepressants exhibit indirect anti-inflammatory properties, their clinical efficacy remains constrained by the challenge of achieving effective concentrations within the CNS and by their limited ability to act on activated microglia. Importantly, these shortcomings do not stem solely from the lack of novel molecular targets, but also from the inherent inability of traditional formulations to selectively and sustainably deliver drugs to such cellular sites. In this context, nanodelivery technologies emerge as particularly relevant.

While the blood–brain barrier (BBB) represents a well-known obstacle across neurological disorders, in MDD, it gains special significance by limiting the penetration of microglia-modulating agents into the brain parenchyma. Consequently, the development of nanosystems should not be regarded merely as a generic strategy to bypass the BBB, but rather as a means of directing pharmacological interventions toward specific neuroimmune circuits, thereby promoting microglial repolarization from the pro-inflammatory M1 to the anti-inflammatory M2 phenotype.

Thus, molecular mechanisms and drug-delivery strategies must be viewed as complementary: understanding the biology of neuroinflammation defines the therapeutic targets (e.g., microglia, cytokines, oxidative stress), whereas nanotechnology provides the pharmaceutic tools to precisely engage them, overcome the heterogeneity of clinical responses, and enhance antidepressant efficacy. The integration of molecular biology and nanotechnology therefore represents a rational and promising pathway toward the development of more effective and personalized therapies for MDD [9,43].

## 5. Brain-Targeted Drug Delivery Systems

The delivery of pharmacological agents to the CNS remains one of the most critical challenges in the treatment of neuropsychiatric disorders, particularly MDD. Despite the growing understanding of the neuroinflammatory mechanisms involved in depression, therapeutic interventions are frequently limited by the restricted accessibility of systemically administered drugs to the brain. This limitation is primarily attributed to the properties of the BBB, a highly specialized interface that regulates molecular exchange between the bloodstream and the brain parenchyma. While the BBB serves a vital homeostatic and neuroprotective role, it also severely limits the brain bioavailability of therapeutic compounds, rendering many potentially effective agents clinically ineffective in CNS disorders, including treatment-resistant depression [44].

Structurally, the BBB is composed of a continuous monolayer of brain microvascular endothelial cells (BMECs), sealed together by tight junctions formed by transmembrane proteins such as claudin-5, occludin, and junctional adhesion molecules, supported intracellularly by scaffolding proteins like ZO-1 [45]. These junctions drastically restrict the paracellular movement of hydrophilic or high-molecular-weight molecules. In contrast to peripheral endothelial cells, BMECs exhibit minimal pinocytosis, complete absence of fenestrae, a high density of mitochondria, and a unique expression pattern of transporters and enzymes, making them uniquely selective [46,47].

The BBB is supported by a complex neurovascular unit composed not only of BMECs but also pericytes, astrocytic end-feet, microglia, the extracellular matrix, and perivascular macrophages. Pericytes, which share the basement membrane with endothelial cells, are particularly abundant in the CNS and play critical roles in microvascular stability, regulation of cerebral blood flow, angiogenesis, and immune surveillance through phagocytic properties. Astrocytes, the most abundant glial cells in the brain, cover approximately 99% of the capillary surface with their end-feet, secreting factors that regulate BBB permeability and participating actively in the control of cerebral metabolism [48,49].

Perivascular macrophages and resident microglia constitute the primary immunological defense at the barrier, capable of responding to systemic or local insults by releasing pro-inflammatory cytokines and reactive oxygen species. In pathological states such as chronic stress or systemic inflammation, excessive activation of these immune components can degrade tight junction proteins, increase BBB permeability, and compromise its selective function [50,51].

The selective permeability of the BBB is governed by both physical and biochemical mechanisms. A major biochemical limitation is the presence of ATP-binding cassette (ABC) efflux transporters, including P-glycoprotein (P-gp/ABCB1) and breast cancer resistance protein (BCRP/ABCG2), which are abundantly expressed on the luminal surface of BMECs. These proteins actively extrude a broad spectrum of structurally diverse drugs from the endothelial cytoplasm back into the bloodstream, limiting intracerebral drug accumulation [52].

Structurally, P-gp is composed of two transmembrane domains and two nucleotide-binding domains that hydrolyze ATP to power the efflux of substrates—primarily lipophilic, neutral, or weakly cationic molecules [53]. BCRP plays a complementary role, particularly in conditions where P-gp is inhibited or saturated, and is more selective for polar and amphiphilic compounds [54].

Importantly, many antidepressants, including fluoxetine, sertraline, citalopram, and venlafaxine, are substrates of these efflux systems. For instance, venlafaxine’s CNS penetration is influenced by P-gp activity, and interindividual variability in treatment response has been linked to genetic polymorphisms in the ABCB1 gene or to drug interactions that modulate transporter expression [55]. This contributes to the heterogeneous clinical outcomes observed in MDD patients undergoing conventional pharmacotherapy [56].

In addition to efflux systems, the BBB also functions as a metabolic barrier equipped with a variety of enzymes that further limit the bioavailability of therapeutics intended for the CNS. Among these enzymes, cytochrome P450 isoforms (e.g., CYP3A4 and CYP2D6), monoamine oxidases (MAO-A and MAO-B), esterases, and peptidases are prominently expressed in BMECs [57,58]. These enzymes catalyze the biotransformation of xenobiotics and endogenous substrates during their transendothelial passage, often resulting in the premature degradation or inactivation of therapeutic agents.

The ability of a drug to cross the BBB is also influenced by its physicochemical properties. Lipophilicity is a major determinant, as lipophilic molecules readily diffuse through the lipid bilayers of endothelial cell membranes. However, other features, such as molecular weight, charge, and hydrogen bonding capacity, also play critical roles. According to Lipinski’s Rule of 5, compounds with high molecular weight (>500 Da), more than five hydrogen bond donors, or more than ten hydrogen bond acceptors, tend to exhibit poor membrane permeability. While originally devised for oral bioavailability, this rule has also been applied, with caveats, to BBB permeability [59,60].

Notably, some antidepressants fit within the favorable physicochemical window (e.g., low molecular weight, moderate lipophilicity, low hydrogen bonding potential), promoting passive diffusion across the BBB. In contrast, drugs classified as “beyond the Rule of 5” may require chemical modifications or carrier-based delivery systems to enhance CNS penetration [61,62]. Carrier-mediated transport (CMT) also plays a vital role in the supply of essential nutrients to the brain. Specific membrane-bound proteins facilitate the transport of glucose (via GLUT1), amino acids, nucleotides, vitamins, and small peptides across BMECs. These transporters operate either by facilitated diffusion or by active transport, using ATP or ionic gradients to mediate substrate influx [63].

Critically, BBB function is dynamic and susceptible to pathological modulation. In MDD, chronic stress, systemic inflammation, and dysregulated cytokine signaling have been implicated in BBB disruption. Preclinical studies report downregulation of claudin-5, increased paracellular permeability, and upregulation of endothelial adhesion molecules such as VCAM-1 and ICAM-1, facilitating peripheral leukocyte infiltration and promoting neuroinflammatory cascades [64,65]. Although this dysfunction may transiently increase drug penetration, it is typically associated with neuronal damage, microglial overactivation, and worsening of depressive pathology, rendering it therapeutically undesirable.

These challenges have encouraged the development of innovative strategies designed to bypass or modulate the BBB. Among these, receptor-mediated transcytosis (RMT) has emerged as a leading approach, whereby nanocarriers are functionalized with ligands targeting overexpressed endothelial receptors, triggering endocytosis and enabling transcellular transport of drug-loaded nanoparticles into the brain parenchyma [66].

**Figure 3:**
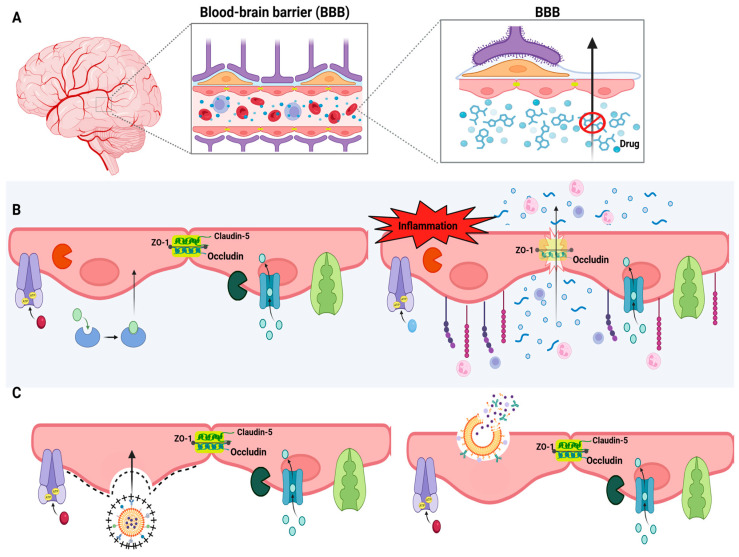
Blood-brain barrier and drug delivery strategies to the CNS. (**A**) Anatomical illustration of the brain and a schematic view of the blood-brain barrier (BBB), highlighting its selective function that restricts drug entry into the brain parenchyma. (**B**) Left panel: Functional BBB structure showing tight junctions composed of proteins such as occludin, claudin-5, and ZO-1, along with efflux transporters (e.g., P-gp) and selective channels. Right panel: BBB dysfunction under inflammatory conditions, leading to junctional disruption, increased paracellular permeability, immune cell infiltration, and compromised barrier selectivity. (**C**) Drug delivery strategies using nanocarriers. Created in BioRender. de Sá Braga Oliveira, A. (2025) https://BioRender.com/yrdy1ld (accessed on 11 December 2025).

Adsorptive-mediated transcytosis (AMT), which leverages electrostatic interactions between cationic surfaces of nanocarriers and negatively charged endothelial membranes, offers a less specific but still viable route of drug delivery [67]. Furthermore, the pharmacological, genetic, or technological modulation of BBB efflux transporters presents a promising complementary strategy, enabling the transient and controlled enhancement of CNS drug delivery (Figure 3).

## 6. Controlled Release Systems

**Figure 4:**
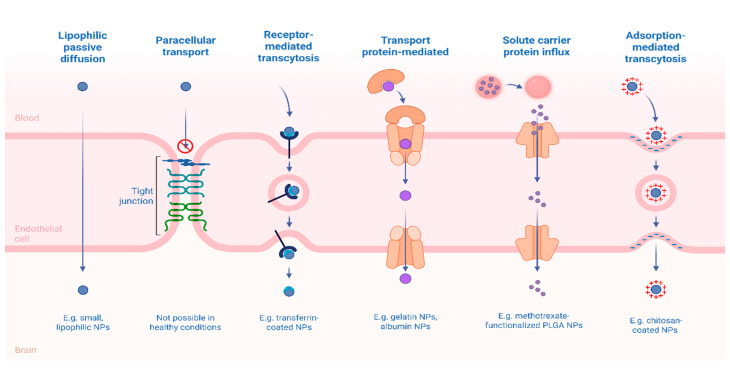
Mechanisms of Transport and Delivery of Nanoparticles Across the BBB. Created in BioRender. Alves, E. (2025) https://BioRender.com/07gtuqp (accessed on 11 December 2025).

In the pursuit of improving therapeutic outcomes for CNS disorders such as MDD, controlled release systems have emerged as transformative tools in neuropharmacology. Unlike conventional formulations that often suffer from rapid clearance, erratic absorption, and non-specific biodistribution, controlled release systems are engineered to deliver therapeutic agents at a predetermined rate, location, and duration [68]. This pharmacokinetic control is particularly relevant in the context of CNS drug delivery, where crossing the BBB is a fundamental obstacle and where fluctuations in drug levels can exacerbate neurotoxicity or diminish efficacy (Figure 4) [69].

Among the most promising technologies in this domain are nanocarriers—engineered systems typically ranging from 1 to 1000 nm, that can be tailored in terms of size, surface properties, composition, and degradation kinetics to optimize delivery to the CNS. Nanoparticles, particularly those based on polymeric and lipidic materials, have shown considerable potential in this regard [70]. Controlled release from these systems is often achieved through diffusion, degradation, swelling, or environmental responsiveness to stimuli such as pH, redox potential, or enzymatic activity. This versatility allows for synchronization of drug release with pathological cues within the CNS microenvironment [71].

In addition to size, nanoparticle morphology plays a crucial role. Rod-shaped nanoparticles demonstrate enhanced adhesion to endothelial surfaces and increased circulation time compared to spherical particles. Particles with sizes ranging between 50 and 100 nm have shown superior ability to cross the BBB [72]. Surface charge is also critical; moderately or highly negative charges (from −1 to −45 mV) are commonly associated with successful BBB permeation, whereas strongly positive nanoparticles may induce BBB toxicity. However, in some cases, cationic surfaces facilitate transport due to electrostatic interactions with the negatively charged glycoproteins present on the BBB [72].

The advantages of controlled release in brain-targeted therapies lie not only in the protection of drugs by encapsulation but also in the capacity of these systems to maintain therapeutic concentrations within the brain over extended periods while minimizing peripheral exposure [68]. This sustained presence is essential not only for drugs with short half-lives or narrow therapeutic indices but also for modulating chronic neuroinflammatory pathways, which are increasingly recognized as contributors to the pathogenesis of MDD [44].

By modulating release kinetics, nanosystems can reduce dosing frequency, enhance patient adherence, and allow for precise spatial-temporal drug targeting. Furthermore, many of these platforms are adaptable to surface functionalization, enabling integration with brain-targeting ligands that exploit receptor-mediated transcytosis [73].

Several ligands have been explored to enhance targeting efficiency, including glycoproteins (e.g., transferrin, lactoferrin), peptides (e.g., glutathione, Angiopeps, cell-penetrating peptides), vitamins (e.g., folate, thiamine), and carbohydrates (e.g., mannose) [74,75]. Surface modification with such ligands not only enhances BBB penetration but also promotes drug accumulation at the target site, reducing off-target toxicity, minimizing dosing, and limiting the development of drug resistance [76].

Among the most widely investigated nanosystems for controlled drug release are PNs, lipid-based systems, and dendrimers. Each platform possesses unique structural and functional properties that can be strategically exploited to optimize brain-targeted therapy. PNs, for instance, are colloidal systems typically ranging from 100 to 500 nm, composed of natural or synthetic biodegradable polymers such as poly (lactic-co-glycolic acid) (PLGA) and chitosan [77].

PLGA is FDA-approved, highly biocompatible, and degrades hydrolytically into lactic and glycolic acid, which are naturally metabolized in the Krebs cycle. Its degradation rate can be precisely tuned by altering the polymer’s molecular weight and the lactic-to-glycolic acid ratio [78]. This tunability makes PLGA nanoparticles particularly attractive for sustained CNS drug delivery, enabling controlled drug exposure over hours to weeks, depending on formulation parameters.

Chitosan, on the other hand, is a natural polysaccharide derived from chitin. It is mucoadhesive, positively charged under physiological conditions, and biodegradable. Chitosan-based nanoparticles are especially useful for intranasal delivery routes targeting the brain, as they can adhere to mucosal surfaces, prolong residence time, and facilitate transport via the olfactory or trigeminal nerves [79]. Furthermore, their amine-rich surface can be easily functionalized with targeting ligands or polyethylene glycol (PEG) to enhance BBB penetration and systemic circulation stability [80].

Dendrimers are nanoscale, highly branched polymers with a globular, tree-like structure. They consist of a central core, an internal branching architecture (called generations), and numerous surface functional groups. These surface termini can be modified with hydrophilic polymers (such as PEG), targeting moieties, or drugs themselves. Internally, dendrimers can encapsulate hydrophobic drugs within their void spaces via hydrophobic or electrostatic interactions, while simultaneously carrying hydrophilic drugs or ligands on their surface. This dual-loading capacity allows for multifunctional drug delivery, combining therapeutic, targeting, and imaging functions in a single system [20].

One of the most studied dendrimer types is poly (amidoamine) (PAMAM) dendrimers, which have well-defined molecular weights and surface chemistries. They offer predictable pharmacokinetics and controlled release based on their generation size and surface modifications [81]. In brain delivery, dendrimers can be designed to cross the BBB through surface conjugation with ligands that exploit receptor-mediated transport pathways. Once in the brain, release is often governed by hydrolysis of cleavable bonds (e.g., ester or disulfide linkages) or by passive diffusion from the dendrimer core [81,82].

Lipid-based nanocarriers also cover a broad category, but solid lipid nanoparticles (SLNs) and nanostructured lipid carriers (NLCs) have garnered particular attention for brain-targeted controlled release. SLNs consist of a solid lipid core stabilized by surfactants, in which the drug is either dispersed or embedded, while NLCs represent a second-generation advancement over SLNs, being composed of a mixture of solid and liquid lipids, creating a less ordered lipid matrix that improves drug loading capacity and reduces drug expulsion during storage [83].

NLCs exhibit superior control over release kinetics due to their semi-crystalline structure and can be optimized to avoid burst release. These lipid particles are particularly suitable for brain delivery because they can be administered via parenteral or intranasal routes, and their lipid composition favors interaction with cell membranes and potential uptake via endocytosis mechanisms [84].

In preclinical models, the theoretical advantages of controlled release systems have translated into significant improvements in brain bioavailability and behavioral outcomes. For example, Dadkhah et al. (2024) demonstrated that fluoxetine-loaded PEGylated chitosan nanoparticles enhanced cognitive performance, increased hippocampal brain-derived neurotrophic factor levels, and reduced demyelination in a rat model of hippocampal injury, outperforming conventional fluoxetine therapy [85].

Similarly, Neves et al. (2021) developed transferrin-functionalized SLN and NLC loaded with curcumin, which significantly enhanced its permeability across an in vitro human BBB model [86]. The functionalized systems demonstrated 1.5-fold higher brain endothelial transport compared to non-functionalized nanoparticles and free curcumin, highlighting their potential for targeted and controlled neuroprotective delivery.

Likewise, Eldeeb et al. (2024) reported that co-administration of chitosan and curcumin nanoparticles not only reversed hydroxyapatite-induced neurotoxicity in rats but also elevated neurotransmitter and antioxidant levels beyond control values: DA by 13% and NE by 24% [87]. While inflammatory markers remained slightly elevated, mitochondrial biogenesis genes were upregulated up to 27% over control, and histological analysis confirmed substantial neuroprotection, underscoring the synergistic potential of these nanosystems in neuroinflammatory conditions.

In summary, controlled-release nanosystems offer a multifaceted solution to the challenges of CNS drug delivery by simultaneously addressing BBB permeability, pharmacokinetic stability, and site-specific release. Depending on the nanocarrier design, these systems can be tailored to overcome key physiological barriers and achieve effective brain targeting. With advancing research and continued clinical validation, the integration of these nanoparticles into therapeutic protocols for MDD and other neurological disorders may substantially improve efficacy and safety profiles compared to conventional drug formulations.

## 7. Drug Delivery Strategies for Microglial Modulation

Microglia are important targets for therapeutic interventions; however, their use as targets presents challenges, such as effectively regulating their states to achieve the desired therapeutic effect. Drug delivery systems can deliver specific modulators to microglia, improving the precision and efficacy of interventions [16]. Different nanocarriers show potential for targeted drug delivery for microglial modulation in the treatment of depression, such as PNs, SLN, magnetic nanoparticles, NLC, hydrogels, self-emulsifying drug delivery systems, and nanoemulsions. Table 1 presents a summary of the information found in the literature about the nanosystems used in the modulation of microglia in the treatment of MDD.

As summarized, differences across platforms become clearer when anti-inflammatory efficacy is judged by concurrent in-vivo microglial involvement and cytokine suppression. Across platforms, the strongest anti-inflammatory signals are observed when in-vivo microglial involvement is demonstrated alongside cytokine suppression. By this criterion, liposomal amphotericin B, curcumin-loaded NLCs, and polymeric memantine nanoparticles show the most consistent profiles (microglia-dependent effects and/or reduced hippocampal ROS/IL-1β/IL-2 with increased IL-10), with polymeric celastrol–minocycline also supporting microglial M1 → M2 repolarization.

SLNs and magnetic nanoparticles with curcumin mainly provide indirect evidence (oxidative-stress/monoamine normalization), whereas nanoemulsions and hydrogels largely show behavioral and neurotransmitter changes without consistent cytokine readouts. Overall, systems with demonstrated microglial targeting/modulation appear more promising for anti-inflammatory action, while studies with standardized endpoints are still needed.

Although several studies included in this review demonstrate a reduction in inflammatory markers and modulation of microglial polarization after the use of nanocarriers, the causal relationship between these effects and behavioral improvement is still considered weak in the literature. This is because many preclinical models evaluate inflammatory and behavioral outcomes in a dissociated manner, without establishing whether the improvement in the tests derives specifically from microglial modulation or from parallel mechanisms, such as increased neurotransmitters, neurogenesis, or reduced oxidative stress. Thus, despite promising evidence, the direct link between nanosystem-directed anti-inflammation and improvement in depressive symptoms remains limited and requires greater methodological standardization, including the simultaneous integration of inflammatory biomarkers, microglial phenotype, and behavior [7,8,106].

Furthermore, different anti-inflammatory agents likely require distinct delivery solutions that align physicochemical constraints with mechanistic goals in the brain. Highly lipophilic and oxidation-prone small molecules (e.g., curcumin, resveratrol, dexanabinol) benefit from lipid-based carriers that enhance solubility, protect from degradation, and improve brain exposure [107]. On the other hand, hydrophilic/cationic small molecules (e.g., memantine) profit from PNs that raise brain/hippocampal accumulation, enable controlled release, and support microglial M1 to M2 modulation [108].

For redox-active small molecules intended to act in activated microglia, dendrimer conjugation (e.g., PAMAM–NAC) affords selective accumulation within activated microglia and attenuation of neuroinflammation [109]. Collectively, these examples support that the same anti-inflammatory endpoint is best achieved by different carriers depending on payload chemistry, target cell state (microglia), and route of administration.

Building on these comparative insights, current scientific evidence indicates that polymeric nanoparticles (PNs) based on poly (lactic-co-glycolic acid) (PLGA), chitosan, or polyethylene glycol (PEG) represent the most promising platforms for microglial targeting in major depressive disorder (MDD). These nanostructures combine high biocompatibility, the ability to cross the blood–brain barrier (BBB), and surface functionalization potential with specific ligands such as transferrin, cell-penetrating peptides, or antibodies facilitating active targeting of activated microglia within neuroinflammatory environments [110,111].

A key mechanism underlying these formulations is the modulation of microglial polarization, promoting the transition from the pro-inflammatory M1 phenotype to the neuroprotective M2 state. This shift is associated with reduced secretion of pro-inflammatory cytokines (TNF-α, IL-1β, IL-6), attenuated oxidative stress, and restoration of synaptic plasticity, thereby improving behavioral and neurochemical outcomes in preclinical models of depression [112].

Preclinical studies demonstrate that functionalized polymeric nanoparticles enhance brain bioavailability of therapeutic agents, improve depressive-like behavior, and exhibit low cytotoxicity even under sustained neuroinflammatory conditions [113]. Compared to other systems, polymeric platforms offer superior physicochemical and pharmacokinetic stability relative to liposomes or nanoemulsions, and lower toxicity than dendrimers or magnetic nanoparticles. Moreover, their potential for intranasal or systemic administration enhances translational feasibility, positioning polymeric nanoparticles as the most balanced and clinically relevant approach for achieving microglia-directed modulation in MDD, with superior efficacy and safety demonstrated in preclinical studies [114,115].

### 7.1. Polymeric Nanoparticles

A variety of nanosystems have been reported to modulate microglial activity. Recent studies have demonstrated that polymeric nanoparticles (PNs) can both inhibit and reprogram the phenotype of these cells, depending on their composition, making them applicable to a range of neurodegenerative diseases, CNS injuries, and MDD [116]. Ns are solid colloidal particles ranging in size from 1 to 1000 nm, obtained from natural, synthetic, or semi-synthetic polymers that can be biodegradable or non-biodegradable.

**Figure 5:**
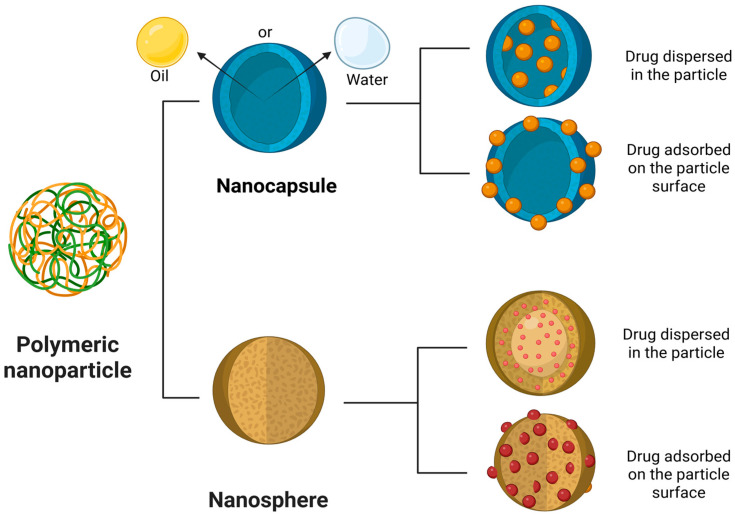
Structure and drug loading approaches in PNs. Schematic representation of the main types of PNs used in controlled drug delivery systems: nanocapsules and nanospheres. Nanocapsules consist of an oily or aqueous core surrounded by a polymeric shell, allowing the drug to be either encapsulated within the core or adsorbed onto the particle surface. Nanospheres are solid matrix systems in which the drug can be homogeneously dispersed throughout the polymeric matrix or adsorbed externally. Created in BioRender. de Sá Braga Oliveira, A. (2025) https://BioRender.com/yrdy1ld (accessed on 11 December 2025).

They can be classified as a nanosphere or a nanocapsule. Nanospheres are matrix particles in which the active pharmaceutical ingredient (API) is adsorbed on the particle surface or dispersed within the particle. On the other hand, nanocapsules are vesicular systems in which the API is confined to a water/oil liquid core surrounded by a polymeric membrane [117,118,119]. PNs can provide controlled and targeted drug release, enhanced drug bioavailability, and an increased therapeutic index [120] (Figure 5).

Jiang et al. (2025) designed memantine-loaded nanoparticles incorporating polydopamine (PDA), a self-adhesive biomimetic polymer, coated with BV2 microglial cell membranes (PDA-Mem@M) [90]. Memantine is an N-methyl-D-aspartate (NMDA) receptor antagonist that reduces brain excitotoxicity, potentially modulating microglial activity. The PDA-Mem@M formulation exhibited a size of 163.5 ± 1.5 nm and a zeta potential of −54.3 ± 2.2 mV, with rapid memantine release under acidic conditions (48% release within 30 min at pH 6.8), relevant to the inflammatory brain microenvironment.

In vitro assays showed no significant cytotoxicity in SH-SY5Y (human neuroblastoma) and bEnd.3 (murine brain endothelial) cells, maintaining over 80% cell viability at high concentrations (200 µg/mL). Additionally, PDA-Mem@M significantly reduced intracellular ROS levels in LPS-induced cellular models and polarized microglia from a pro-inflammatory M1 phenotype (reduction in CD86, TNF-α, and IL-2) to an anti-inflammatory M2 phenotype (increase in CD206, TGF-β, and IL-10) in BV2 cells.

In vivo studies over 21 days in mice subjected to a chronic unpredictable stress (CUS) model demonstrated that PDA-Mem@M exhibited enhanced brain and hippocampal targeting (1.3-fold higher fluorescence than PDA) after 4 h post-injection. PDA-Mem@M outperformed memantine monotherapy at lower and less frequent doses, indicating a faster onset of action and greater efficacy, with minimal observed toxicity. Furthermore, the nanoformulation reduced hippocampal ROS and pro-inflammatory cytokines (IL-1β and IL-2), while increasing anti-inflammatory cytokines (IL-10), stimulating neurogenesis, and exerting neuroprotective effects by reducing neuroinflammation and restoring synaptic plasticity [90].

Beyond synthetic polymeric systems, endogenous carriers like exosomes offer another promising approach to targeted microglial modulation due to their inherent biocompatibility and natural ability to cross the BBB. They have been employed in nanoformulations to deliver molecules like celastrol and minocycline (anti-inflammatory and neuroprotective agents) across the BBB to resident brain cells [121].

Researchers developed PLGA nanoparticles loaded with exosomes, celastrol, and minocycline (CMC-EXPL), exhibiting a size of 132 nm and a zeta potential of −35.5 mV [91]. In BV2 microglial cells, the nanoformulation achieved 65.8% uptake within 270 min. Cocultures of SH-SY5Y neurons and LPS-stimulated BV2 microglia (induced to an M1 phenotype) revealed that CMC-EXPL significantly suppressed M1 polarization (reduction in CD80 and iNOS) and promoted M2 polarization (increase in CD206 and Arg1), showing superior anti-inflammatory therapeutic potential compared to other tested formulations.

The animal model of post-stroke depression (POSD), induced by middle cerebral artery occlusion (MCAO) followed by chronic unpredictable mild stress (CUMS), was used for CMC-EXPL administration. The nanoformulation significantly attenuated weight loss and reversed depressive-like behaviors. M1 microglial markers (iNOS and CD86) were elevated in MCAO and POSD groups, while M2 markers (Arg-1 and CD206) were reduced. Treatment with CMC-EXPL significantly downregulated M1 markers and upregulated M2 markers, indicating enhanced microglial polarization toward the M2 phenotype in POSD rats, contributing to its antidepressant efficacy [91]. This strategy not only enhanced brain delivery but also effectively reprogrammed microglia in complex models, suggesting broader applicability to comorbid neuropsychiatric conditions.

In the study by Nagpal, Singh, and Mishra (2013), the safety and efficacy of chitosan-based PNs encapsulating minocycline (MHPNs) were evaluated. In vivo results demonstrated that MHPNs exhibited antidepressant effects and were safe, with no observed toxicological effects on the brain, heart, spleen, liver, or kidneys [122].

Similarly, Yusuf et al. (2016) incorporated curcumin into PNs (CUR-PNs), which showed enhanced antidepressant activity in the Forced Swim Test (FST) and Tail Suspension Test (TST), even at a low dose (5 mg/kg) [88]. CUR-PNs increased brain bioavailability by 1.6 times compared to free curcumin. The in vitro release profile was biphasic, with an initial release of 69.43 ± 6.56% at 24 h and sustained release reaching 95.56 ± 4.67% at 144 h, following the Higuchi model (r^2^ = 0.95). CUR-PNs also significantly increased the activity of antioxidant enzymes SOD and catalase (CAT), and exhibited strong interaction with MAO-B (ΔG = −7.79 kcal/mol), suggesting an antidepressant effect through multiple mechanisms. These findings highlight the potential of CUR-PNs for controlled release, improved brain penetration, and enhanced therapeutic efficacy.

Yang et al. (2024) investigated the effects of selenium nanoparticles (SePNs) in alleviating depression-like behavior induced by fluoride (F) exposure in mice, focusing on the JAK2–STAT3 signaling pathway [78]. In vivo results showed that SePNs restored DA and NE levels in the cortex, reduced microglial activation and interleukin-1β secretion, inhibited the nuclear translocation of p-STAT3, increased the number of surviving neurons, and reduced cortical vacuolization. These outcomes indicate that SePNs mitigated fluoride-induced neurotoxicity and depressive-like behaviors, possibly by inhibiting the JAK2–STAT3 pathway.

Zhu et al. (2021) evaluated the antidepressant effects of polydopamine nanoparticles (PDA-PNs, 2.5 mg/kg) in C57BL/6 mice with LPS-induced depression (1 mg/kg, i.p.). PDA-PNs reversed depressive and anxious behaviors, reducing immobility in the TST from 170.8 ± 10.6 s to 95.2 ± 11.6 s and increasing center time in the open field test from 38.7 ± 4.3 s to 68.7 ± 6.2 s. Additionally, PDA-PNs significantly lowered TNF-α levels from 235.8 ± 20.4 to 136.5 ± 9.4 pg/mg and IL-1β from 143.2 ± 17.3 to 83.9 ± 5.9 pg/mg. The PN also reduced splenomegaly, inhibited microglial activation (Iba1), downregulated IL-1β and iNOS expression, upregulated Arg-1, crossed the blood-brain barrier, and reduced brain oxidative stress, thereby demonstrating a neuroprotective effect [123].

### 7.2. Dendrimers

Dendrimers are hyperbranched, radially symmetric macromolecules with a precisely tailored architecture composed of surface functional groups. These surface functional groups can be modified to alter their physicochemical and biological properties [124]. In another study, PAMAM dendrimers labeled with Cyanine5 (D-Cy5) were used to investigate microglial migration dynamics and dendrimer interactions in the presence of neuroinflammation. Using organotypic whole-hemisphere brain slice cultures from newborn rabbits, PAMAM uptake (4 nm in size) by microglia was faster and more pronounced in slices from rabbits with cerebral palsy (CP) than in healthy controls (1.6-fold increase after 4 h, with 80% of microglia containing dendrimers).

In vivo studies demonstrated selective accumulation of PAMAM dendrimers in activated microglia in the brains of newborn rabbits. When conjugated with N-acetylcysteine (NAC), the formulation significantly improved motor function and attenuated neuroinflammation. These findings suggest that increased dendrimer uptake by impaired microglia can be exploited for targeted drug delivery and functional modulation [105].

### 7.3. Lipid-Based Nanoparticles

SLNs are particles with a size of <1000 nm, which have a solid hydrophobic core of biocompatible and biodegradable lipids such as triglycerides, partial glycerides, waxes, steroids, and fatty acids that are solid at normal room temperature. These lipids are stabilized by different biocompatible surfactants that can be ionic or nonionic. SLN offers a viable method for the incorporation of hydrophilic or lipophilic drugs and peptides, enhanced drug targeting and bioavailability, controlled drug release, enhanced drug stability, and increased drug payload [125].

Similarly, lipid-based nanosystems such as NLCs have been widely studied for brain delivery due to their favorable size, biocompatibility, and ability to traverse biological membranes [98]. Thus, NLCs loaded with curcumin (CUR-NLCs) were investigated for their neuroprotective effects in a rat model of LPS-induced depression and anxiety. CUR-NLCs exhibited a particle size of 147.8 ± 10.4 nm, surface charge of −32.8 ± 1.4 mV, and an encapsulation efficiency of 91.0 ± 4.6%.

In vivo studies indicated an indirect microglial effect, as CUR-NLCs markedly attenuated LPS-induced neurodegeneration, restored tissue architecture and cellular integrity, and increased neuronal survival. Additionally, CUR-NLCs suppressed brain expression of pro-inflammatory markers such as p-NF-κB, TNF-α, and COX-2, thus reducing neuroinflammation [97]. Although the microglial effects were indirect, these results underscore the anti-inflammatory potential of natural compounds like curcumin when properly encapsulated for CNS delivery.

Building on these findings, other antidepressants such as agomelatine have also been encapsulated into NLCs to enhance therapeutic response through inflammatory pathway modulation. For instance, Gul et al. (2022) developed agomelatine-loaded NLCs (AGM-NLCs) with an average size of 99.8 ± 2.6 nm and a charge of 23.2 ± 1.2 mV to enhance in vivo antidepressant potential. Agomelatine supports neuronal integrity and exerts an indirect effect on microglia by reducing inflammation. AGM-NLCs effectively suppressed LPS-induced neuroinflammation, reducing both expression and concentration of TNF-α and COX-2 in the brain [99].

Rubab et al. (2021) produced curcumin encapsulated nanostructured lipid carriers (CUR-NLCs) with a particle size of 147.8 ± 10.4 nm, PDI of 0.27 ± 0.02, zeta potential of −32.8 ± 1.4 mV, and incorporation efficiency of 91.0 ± 4.6%. In vitro release study demonstrated ~27% drug release within 2 h, followed by sustained release with ~54% and ~73% curcumin release within 12 h and 24 h, respectively. The results of in vivo studies in rats subjected to a lipopolysaccharide-induced model of depression and anxiety revealed a significant increase in fighting time (95.3 ± 5.1 s) and a reduction in immobility time (87.9 ± 4.3 s) in the forced swimming and tail suspension tests. In addition, there was a substantial increase in the time spent (65.0 ± 2.7 s) and in the number of entries into the illuminated and open compartments (14.7 ± 0.7 s) in the light-dark box and elevated plus maze tests, indicating antidepressant and anxiolytic effects. Histological and immunohistochemical analyses revealed a reduction in brain inflammation with suppression of the expression of inflammatory markers such as p-NFκB, TNF-α, and COX-2 in brain tissue [97].

Liposomes, on the other hand, are spherical lipid bilayer vesicles composed primarily of phospholipids. Their structures result from the self-assembly of amphiphiles in an aqueous medium, forming single or multiple concentric bilayers, where the polar head groups are in contact with the aqueous medium and the fatty acids form the hydrophobic core of the bilayers that are protected from water. They present several advantages, such as biocompatibility, biodegradability, low toxicity, non-immunogenicity, improved drug solubility, the possibility of co-encapsulation, and surface modifications for targeted, controlled, and sustained release of payload materials [119,126].

Interestingly, even existing drugs not originally developed for depression have shown antidepressant effects when incorporated in nanocarrier systems, particularly via microglial activation. For example, Gao et al. (2019) investigated the antidepressant potential of commercially available liposomal amphotericin B (L-AmB). Male mice (6–8 weeks old) subjected to CUS or LPS-induced depression were treated with either free AmB (1, 3, or 5 mg/kg, i.p.) or liposomal AmB (0.5, 1, or 3 mg/kg, i.p.). In CUS-exposed mice, L-AmB (1 and 3 mg/kg) dose-dependently reversed depressive-like behaviors—decreased immobility in the Tail Suspension Test (TST) and Forced Swim Test (FST), increased sucrose consumption in the Sucrose Preference Test (SPT), and increased center time in the Open Field Test (OFT)—effects not observed with free AmB. Regarding microglial activation, pretreatment with minocycline (a microglial inhibitor) suppressed the antidepressant effect of L-AmB in CUS-stressed mice, with reduced IL-1β and IL-6 levels. Similarly, PLX3397, a microglial depletor, abolished both the antidepressant and pro-inflammatory effects of L-AmB in treated groups. These findings confirm that modulating microglial activation, rather than simply suppressing it, may be essential to the antidepressant action of certain compounds [102].

### 7.4. Inorganic Nanoparticles

Magnetic nanoparticles (MNPs) are nanometer-sized materials that respond to external magnetic fields. They are typically composed of iron oxides such as magnetite (Fe_3_O_4_) or maghemite (γ-Fe_2_O_3_), pure metals like Fe, Co, or Ni, or a combination of metals and polymers. These particles may be coated with polymers, lipids, or silica to enhance their stability, biocompatibility, and functionalization. The magnetic behavior of these materials can be tuned not only by external magnetic fields but also by their size, shape, and morphology [127].

Khadrawy et al. (2021) developed iron oxide nanoparticles coated with curcumin (Cur-IONPs) with an average size of 15 ± 3 nm and a zeta potential of −25.4 mV, indicating good stability. In a reserpine-induced rat model of depression, treatment with Cur-IONPs (5 mg/kg for 14 days) significantly reduced immobility time in the forced swim test, restoring active swimming time to near-control levels, suggesting behavioral improvement. Markers of oxidative stress, which were elevated in the cortex (MDA +48.9%, NO +55%, GST +61%, GSH −29%) and hippocampus (MDA +36.1%, NO +233%, GST +29.8%, GSH −16.9%), were normalized after treatment, indicating an antioxidant effect. Enzymatic activities altered in the cortex (AchE +144.7%, Na^+^/K^+^-ATPase +18.2%, MAO +56.6%) and hippocampus (AchE +52.1%, Na^+^/K^+^-ATPase +24.2%, MAO +92.5%) were also restored to values close to those of the control group, suggesting improved neuronal function and neurotransmitter regulation. Regarding neurotransmitter levels, reductions observed in the cortex (5-HT −37.8%, NE −65.9%, DA−49.6%) and hippocampus (5-HT −46.6%, NE −40.7%, DA −32%) were partially reversed, with noradrenaline and DA levels restored in the cortex and 5-HT in the hippocampus, corroborating the antidepressant effects of the treatment [95].

In the reserpine-induced depression model studied by Fahmy et al. (2024), there was a significant reduction in behaviors such as square crossing frequency (−100%), rearing (−100%), and grooming (−84.75%), along with a 663.3% increase in immobility time and a 91% reduction in struggle time. Treatment with Zn(cur)O and ZnO nanoparticles, as well as free curcumin, attenuated these effects. Grooming behavior increased by up to 90.25% with curcumin and 59.73% with ZnO PNs; immobility time was reduced to 228.9% (Zn(cur)O NPs) and 50% (ZnO PNs). MDA levels were restored to control values, and GSH levels in the cortex, hippocampus, and striatum significantly increased (up to 384.7% with Zn(cur)O NPs). Levels of the neurotransmitters 5-HT and NE were significantly elevated, with hippocampal 5-HT increasing by 146.17% following treatment with Zn(cur)O PNs, indicating a strong antidepressant effect of these compounds [96].

Fahmy et al. (2024) further investigated the antidepressant activity of zinc oxide nanoparticles conjugated with curcumin (ZnO PNs) in adult male Wistar rats with reserpine-induced depression. The nanoparticles had a particle size of 342 ± 22.3 nm and a zeta potential of −25.6 ± 4.61 mV. In vivo results showed improved motor activity and reduced immobility time. Additionally, a decrease in MDA levels and an increase in reduced glutathione (GSH) and CAT levels were observed, along with elevated concentrations of 5-HT and NE [96].

## 8. Future Perspective

Despite consolidated studies involving nanoparticles and microglial inflammation, their application in the treatment of MDD remains underexplored. Therefore, it is necessary to develop increasingly sophisticated therapeutic systems capable not only of crossing the BBB but also of selectively responding to the specific inflammatory microenvironment associated with depression. Such strategies could overcome the limitations of conventional antidepressants, offering greater efficacy, fewer side effects, and improved treatment adherence.

One of the most promising approaches is functionalization for selective targeting of hyperactivated microglia. Ligands such as transferrin [128] and mannose can be employed to exploit receptors expressed in activated microglia, thereby enhancing nanoparticle internalization [129]. In addition, these systems can be designed to respond to the microenvironment through pH changes in inflamed regions, excessive ROS, or specific enzymes such as myeloperoxidase and matrix metalloproteinases [130]. In this way, encapsulated molecules with anti-inflammatory or antioxidant activity could be locally released, reprogramming microglia from the pro-inflammatory M1 phenotype to the anti-inflammatory M2 phenotype.

Another notable front involves theragnostic nanosystems, which integrate both therapeutic and diagnostic functions. Beyond drug delivery, these platforms can be functionalized with imaging agents, such as magnetic iron oxide nanoparticles (for magnetic resonance imaging) or fluorescent dyes traceable by bioimaging techniques [131]. This enables real-time monitoring of neuroinflammation by detecting microglial marker expression or oxidative stress intensity, correlating these findings with clinical responses. Such dynamic monitoring may allow for individualized adjustments in therapy.

Furthermore, the co-encapsulation of molecules with complementary activities emerges as a relevant strategy to simultaneously address different MDD targets [132]. Natural compounds such as curcumin and resveratrol have well-documented anti-inflammatory and antioxidant effects, in addition to potential antidepressant properties [133]. Meanwhile, agomelatine and ketamine exhibit established antidepressant activity with indirect effects on inflammation and neuroplasticity [134]. Controlled combination of these agents within nanosystems could generate synergistic effects, reducing required doses and minimizing toxicities.

Additionally, administration of nanoparticles through alternative routes, such as intranasal delivery, enables direct transport of these nanosystems to the central nervous system, bypassing the BBB and reducing systemic side effects [135]. Biomimetic systems, such as exosomes or nanoparticles coated with cellular membranes, also stand out for enhancing biocompatibility, promoting immune evasion, and mimicking physiological pathways of cell communication [136].

Despite these advances, it is crucial to emphasize that safety concerns and translational barriers remain major obstacles to clinical application. Nanoparticles can induce inflammatory responses, oxidative stress, genotoxicity, and mitochondrial dysfunction depending on their composition, size, and surface chemistry, highlighting the need for rigorous toxicological assessment. Moreover, their ability to cross physiological barriers raises uncertainties regarding long-term accumulation, off-target interactions, and potential neurotoxicity. Overcoming these limitations will require integrated efforts to ensure biocompatibility, reproducibility, and robust characterization of nanosystems before they can be safely incorporated into pharmacotherapy for MDD [137,138].

In summary, nanoparticles must evolve toward greater selectivity and responsiveness, combining multiple therapeutic mechanisms while enabling real-time monitoring of treatment outcomes. By integrating nanotechnology with pharmacogenomics and inflammatory biomarkers, there is potential to achieve personalized and more effective therapies for patients with treatment-resistant depression. This path represents not only a technological innovation but also a paradigm shift in microglial modulation as a clinical strategy against MDD.

## 9. Conclusions

MDD remains a major global health challenge, largely due to the multifactorial nature of its pathophysiology and the limited efficacy of current pharmacological treatments. Emerging evidence strongly supports the role of microglial activation and neuroinflammation as key drivers of treatment resistance, highlighting the need for therapeutic strategies that go beyond monoaminergic modulation. Nanotechnology offers a unique opportunity to address these challenges by enabling targeted drug delivery, controlled release, and co-delivery of multimodal agents capable of modulating microglial function and in-flammatory signaling pathways.

Preclinical studies have shown that nanocarriers can improve antidepressant bioavailability, enhance brain penetration, and promote neuroprotective effects through antioxidant and anti-inflammatory actions. While clinical translation remains in its early stages, the integration of nanotechnology with precision medicine approaches holds promise for the development of next-generation therapies for depression. Targeting microglia via nanotechnology emerges as a cutting-edge strategy with the potential to transform the therapeutic landscape of MDD, offering more effective and personalized interventions.

## Figures and Tables

**Table 1:** Nanosystems used for modulation of microglia in the treatment of MDD.

Drug Delivery Systems	Compound Used	Physicochemical Parameters	In Vitro Activity	In Vivo Activity	Key Mechanism Related to Microglia/Inflammation	Reference
Polymeric nanoparticle	Curcumin	194.3 ± 14.8 nm. 19.5 ± 2.6 mV. Drug release: 83.78 ± 7.43 to 95.56 ± 4.67% in 144 h	NA	↓ immobility (FST/TST), ↑ SOD/catalase activity	NF-κB inhibition; ↓ TNF-α/↓ IL-1β; antioxidant activity (↑SOD/CAT) reducing microglial activation.	Yusuf (2016) [88]
Polymeric nanoparticle	Dopamine hydrochloride	244 nm −48 mV	≥90% DPPH scavenging at 20 µg/mL; ↓ ROS in LPS-challenged PC12 cells	NA	Reduction of ROS; protection against LPS-induced oxidative stress; attenuation of pro-inflammatory signaling in microglia.	Zhang (2023) [89]
Polymeric nanoparticle	Memantine	163.5 ± 1.5 nm −54.3 ± 2.2 mV	↓ ROS; polarized microglia from M1 to M2; ↓ CD86, TNF-α and IL-2; ↑ CD206, TGF-β, IL-10	↑ Brain/hippocampal uptake (≈1.3× vs. PDA); >memantine monotherapy at lower/less-frequent dosing; minimal toxicity; ↓ ROS/IL-1β/IL-2; ↑ IL-10; ↑neurogenesis; restored synaptic plasticity/neuroprotection.	NMDAR blockade/modulation; ↓ ROS; M1 → M2 reprogramming (↓ CD86, TNF-α; ↑ CD206, IL-10) and neuroprotective effects.	Jiang et al. (2025) [90]
Polymeric nanoparticle	Celastrol and minocycline	132.0 nm −35.5 mV	M1 → M2 shift in LPS-BV-2: ↓ CD80/iNOS; ↑ CD206/Arg1; superior anti-inflammatory efficacy vs. other nanoformulations.	Reversed depressive-like behavior and attenuated weight loss in POSD rats;↓ iNOS/CD86, ↑ Arg-1/CD206 (M1 → M2), consistent with antidepressant efficacy	Induction of M1 → M2 (↓ iNOS/CD80, ↑ Arg-1/CD206); suppression of pro-inflammatory cytokines; inhibition of MAPK/NF-κB pathways.	Lv et al. (2024) [91]
Solid lipid nanoparticle	Curcumin	291 to 312 nm 22–36 mV	NA	Reversed the effects of Aβ_25–35_ (↑ 663.3% immobility in TST/FST), normalized SOD/CAT levels	Antioxidant effect + SOD/CAT normalization; indirect suppression of inflammatory markers → reduction of microglial activation.	Fidelis et al. (2019) [92]
Solid lipid nanoparticle	Curcumin and dexanabinol	−22.6 ± 0.9 mV	↑ DA/5-HT; reduction in cellular apoptosis	↑ DA/5-HT levels and mRNA expression of CB1, p-MEK1, and p-ERK1/2 in the hippocampus and striatum	Increased monoamines (DA/5-HT) and reduced apoptosis; modulation of CB1 pathways → indirect impact on microglia and inflammation.	He et al. (2017) [93]
Solid lipid nanoparticles	HU-211 and curcumin	−21.7 ± 0.4 mV Drug release: 77% in 7 days	↑ expression of CB1, p-MEK1, and p-ERK1/2; cellular uptake: 99%	↑ DA levels	Activation of MAPK/ERK signaling (p-MEK1/p-ERK1/2) and increased DA; reduction of neuronal stress with an indirect anti-inflammatory effect.	He et al. (2016) [94]
Magnetic nanoparticles	Curcumin	15 ± 3 nm −25 mV	NA	↑ in Na^+^, K^+^-ATPase activity and ↑ levels of monoamine neurotransmitters. Prevention of excitotoxicity mediated by NMDA receptor overactivation	Improved enzymatic activity (Na^+^/K^+^-ATPase) and increased monoamines; reduced excitotoxicity and indirect anti-inflammatory effect on microglia.	Khadrawy et al. (2021) [95]
Magnetic nanoparticles	Curcumin	342 ± 22.3 −25.6 ± 4.61 mV	NA	↓ malondialdehyde, ↑ reduced glutathione (GSH) and catalase, and elevated concentrations of 5-HT and NE	Reduction of lipid peroxidation (↓ MDA), ↑ GSH/CAT; normalization of 5-HT and NE, antioxidant effects that attenuate microglial activation.	Fahmy et al. (2024) [96]
Nanostructured lipid carriers	Curcumin	147.8 ± 10.4 nm −32.8 ± 1.4 mV Drug release: ~54% and ~73% at 12 h and 24 h. respectively	NA	Suppression of p-NF-κB, TNF-α, and COX-2 expression	Suppression of p-NF-κB, ↓ TNF-α and ↓ COX-2 (in vivo), direct anti-inflammatory action in brain tissue.	Rubab et al. (2021) [97]
Nanostructured lipid carriers	Curcumin	147.8 ± 10.4 nm −32.8 ± 1.4 mV	NA	↓ LPS-induced neurodegenerative damage, ↑ tissue architecture and cellular integrity; ↓ p-NF-κB, TNF-α, and COX-2 induced by LPS in the brain	Protection against LPS-induced neurodegenerative damage; ↓ p-NF-κB, TNF-α, COX-2—reduction of neuroinflammation.	Zeb et al. (2020) [98]
Nanostructured lipid carriers	Agomelatine	99.8 ± 2.6 nm 23.2 ± 1.2 mV	NA	↓ LPS-induced neuroinflammation, TNF-α, and COX-2	Suppression of LPS-induced inflammation (↓ TNF-α, ↓ COX-2); indirect anti-inflammatory effect mediated by improved neuronal signaling.	Gul et al. (2022) [99]
Hydrogel	Resveratrol	Res ~35% and Res-THS ~59% in 10 h	NA	Immobility time in the FST (~180 s → ~95 s); Corticosterone levels (~160 → ~100 ng/mL); 5-HT, DA, and NA levels in the brain.	Antioxidant action + reduction of the HPA axis (↓ corticosterone); normalization of monoamines → anti-inflammatory effect and reduction of microglial activation.	Zhou et al. (2022) [100]
Hydrogel	Curcumin	55.54% release in 10 h.	NA	↓ immobility time in the forced swim test and tail suspension test; reversal of ptosis and hypothermia; increased levels of 5-HT, NE, and DA in the brain; effect comparable to fluoxetine	↓ Immobility (FST/TST) with increased monoamines; antioxidant/anti-inflammatory effect that contributes to decreased microglial activation.	Qi et al. (2020) [101]
Liposome	Amphotericin B	NA	NA	↓ depressive behaviors in a dose-dependent manner (not observed with isolated drug)	Activation/modulation of microglia is necessary for the antidepressant effect (minocycline/PLX3397 blocks the effect); microglia-dependent mechanism (↑ IL-1β/IL-6 modulation).	Gao et al. (2019) [102]
Nanoemulsion	Curcumin	116.0 ± 0.30 nm −11.6 ± 1.23 mV Drug release: 36.51 ± 3.24% release in HCl and 44.90 ± 2.47% in PBS over 48 h	TUR exhibited the highest antioxidant activity (ABTS•^+^), followed by Curcumin and Vitamin C, with IC_50_ values of 17.9, 29.1, and 57 µg/mL, respectively	TUR-NE (CUMS): ↑ Sucrose preference (77.5% vs. 54.0%); ↓ FST immobility (*p* < 0.01); ↓ NSFT latency; ↑ 5-HT (plasma 21.7 vs. 16.3 ng/mL; brain 46.9 vs. 44.0 ng/mL)	Increased preference for sucrose and ↓ FST; systemic antioxidant/anti-inflammatory profile that reduces pro-inflammatory brain signals.	Sheng et al. (2023) [103]
Self-emulsifying system	Curcumin	150 nm Drug 100% released within 5 min	NA	7.4 ± 0.2 mL (sucrose); 103.4 ± 5.8 s (FST); 247 ± 3.1 g (weight); Partial neuroprotective and hepatoprotective effects	Fast delivery and increased bioavailability of curcumin → antioxidant and anti-inflammatory effects with reduced microglial activation.	Suchiwa Pan et al. (2023) [104]
Dendrimers	Polyamidoamine	4 nm	microglia took up PAMAM dendrimers faster and to a greater extent than healthy controls (~1.6× higher at 4 h; ~80% of microglial cells contained dendrimers)	Selective accumulation in activated microglia; ↑ motor function; ↓ neuroinflammation	Selective accumulation in activated microglia; targeted delivery (PAMAM–NAC) → direct reduction of neuroinflammation and functional improvement via microglial modulation.	Zhang et al. (2016) [105]

NA: The study was not applied or performed in the article. ↑ increase ↓ decrease.

## Data Availability

No new data were created or analyzed in this study.
